# Enhancement of therapeutic potential of a naturally occurring human antibody targeting a phosphorylated Ser^422^ containing epitope on pathological tau

**DOI:** 10.1186/s40478-018-0562-9

**Published:** 2018-07-12

**Authors:** Jeroen van Ameijde, Rosa Crespo, Roosmarijn Janson, Jarek Juraszek, Berdien Siregar, Hanneke Verveen, Imke Sprengers, Tariq Nahar, Jeroen J. Hoozemans, Stefan Steinbacher, Roland Willems, Lore Delbroek, Marianne Borgers, Koen Dockx, Kristof Van Kolen, Marc Mercken, Gabriel Pascual, Wouter Koudstaal, Adrian Apetri

**Affiliations:** 1 Janssen Prevention Center, Janssen Pharmaceutical Companies of Johnson and Johnson, Archimedesweg 6, 2333 Leiden, CN the Netherlands; 2 Department of Pathology, Amsterdam Neuroscience, VU University Medical Center, De Boelelaan 1117, 1081 HV Amsterdam, the Netherlands; 3 Proteros Biostructures GmbH, Bunsenstraße 7a, 82152 Planegg, Germany; 4 Janssen Neuroscience Discovery, Janssen Pharmaceutical Companies of Johnson and Johnson, Turnhoutseweg 30, 2340 Beerse, Belgium; 5 Molecular and Cellular Pharmacology, Discovery Sciences, Janssen Pharmaceutical Companies of Johnson and Johnson, Turnhoutseweg 30, 2340 Beerse, Belgium; 6 Janssen Prevention Center, Janssen Pharmaceutical Companies of Johnson and Johnson, 3210 Merryfield Row, San Diego, CA 92121 USA

**Keywords:** Tau, Nucleation, Aggregation, Phosphorylation, Monoclonal antibody, Intervention

## Abstract

Aggregation of tau protein and spreading of tau aggregates are pivotal pathological processes in a range of neurological disorders. Accumulating evidence suggests that immunotherapy targeting tau may be a viable therapeutic strategy. We have previously described the isolation of antibody CBTAU-22.1 from the memory B-cell repertoire of healthy human donors. CBTAU-22.1 was shown to specifically bind a disease-associated phosphorylated epitope in the C-terminus of tau (Ser^422^) and to be able to inhibit the spreading of pathological tau aggregates from P301S spinal cord lysates in vitro, albeit with limited potency. Using a combination of rational design and random mutagenesis we have derived a variant antibody with improved affinity while maintaining the specificity of the parental antibody. This affinity improved antibody showed greatly enhanced potency in a cell-based immunodepletion assay using paired helical filaments (PHFs) derived from human Alzheimer’s disease (AD) brain tissue. Moreover, the affinity improved antibody limits the in vitro aggregation propensity of full length tau species specifically phosphorylated at position 422 produced by employing a native chemical ligation approach. Together, these results indicate that in addition to being able to inhibit the spreading of pathological tau aggregates, the matured antibody can potentially also interfere with the nucleation of tau which is believed to be the first step of the pathogenic process. Finally, the functionality in a P301L transgenic mice co-injection model highlights the therapeutic potential of human antibody dmCBTAU-22.1.

**Electronic supplementary material:**

The online version of this article (10.1186/s40478-018-0562-9) contains supplementary material, which is available to authorized users.

## Introduction

Aggregated tau protein is associated with a range of neurological disorders, including Alzheimer’s disease [31]. Tau is predominantly expressed in neurons where, in its native state, it facilitates axonal transport and cytoskeletal remodeling by interacting with tubulin [17, 19]. This interaction is thought to be modulated through regulated phosphorylation of some of the many potential phosphorylation sites present in tau [6, 39]. However, under pathogenic conditions tau becomes hyperphosphorylated and aggregates into paired helical filaments (PHFs) and insoluble neurofibrillary tangles (NFTs) which impair axonal transport and synaptic function and ultimately lead to cell death (reviewed in [26]). The aggregation of tau is believed to follow a nucleation dependent polymerization (NDP) process [2, 16, 22, 45], characterized by an initial nucleation step followed by an exponential growth. Accumulating evidence indicates that misfolded tau aggregates released from neurons can be taken up by synaptically connected neurons and cause normal tau in the recipient neurons to aggregate, resulting in spreading of tau pathology trough neuronal connectivity [13, 21, 24, 29, 40]. This propagation through extracellular transfer of tau between cells suggests tau immunotherapy may be a viable therapeutic strategy for tauopathies. Indeed, promising results have been reported for both active and passive immunization targeting tau in animal models [3, 5, 7, 43, 44, 47, 50, 51].

We have previously described the isolation of a panel of monoclonal antibodies directed against pathological tau structures from the memory B cell repertoire of apparently healthy individuals [35]. One of these antibodies, CBTAU-22.1, was shown to recognize the C-terminus domain of tau, its binding being dependent on phosphorylation of Ser^422^. Phospho-Ser422 is not observed on tau under physiological conditions, but only on pathological tau and associated with numerous neurodegenerative disorders [4, 10, 12, 23, 41]. In line with this notion, CBTAU-22.1 detects pathological tau structures in AD, PART, PSP, FTDP-17 and Pick’s disease, but does not stain healthy brain tissue [35]. Using a cell-based biosensor assay adapted from Yanamandra et al. [51], we previously showed that CBTAU-22.1 is able to inhibit seeding activity of PHFs from P301S spinal cord lysates, suggesting that this fully human antibody might be able to slow the propagation of tau pathology in vivo [35]. However, its in vitro inhibitory effect was low, i.e. compared to that of murine anti-PHF antibody AT8 [32], which would likely limit any therapeutic potential. We hypothesized that this relatively low potency was caused by a relatively low affinity for PHFs. Here we used a combination of rational and random approaches to derive a variant antibody with improved affinity and assessed its specificity as well as its functionality.

## Materials and methods

### Expression, purification and analysis of recombinant IgG and tau

Human IgG1, chimeric IgG2a antibodies, Fab fragment, as well as recombinant tau were constructed, produced and purified in the same manner as previously described [1]. After purification, the antibodies as well as 2N4R-tau were characterized by size exclusion chromatography coupled with multi angle light scattering (SEC-MALS) by in-line coupled detectors on a TSKgel G3000SWxl gel filtration column **(**Tosoh Bioscience) equilibrated with 150 mM sodium phosphate, 50 mM sodium chloride pH 7.0 at a flow rate of 1 mL/min. The following detectors were used: UV (Agilent 1260 Infinity MWD, Agilent Technologies), refractive index (Optilab T-rEX, Wyatt Technology) and 8-angle static light scattering (DAWN HELEOS 8+, Wyatt Technology). The molar mass as well as the monomeric status of the samples were assessed and if samples contained > 5% of aggregates or fragments they were gel filtrated using a Superdex HiLoad 26/60 Superdex 200 pg column (GE Healthcare). The antibodies were furthermore analyzed by SDS-Page and their functionality confirmed by binding to cognate tau peptide V1089–24 (Pepscan, The Netherlands), sequence SSTGSIDMVD(pS)PQLATLA corresponds to residues 412–429 of tau with phosphorylation at Ser^422^ (Additional file 1: Table S2), by Octet biolayer interferometry (Octet Red 384, ForteBio).

### Qualitative association and dissociation measurements by octet biolayer interferometry

Biolayer interferometry (Octet Red 384, ForteBio). was used to screen CBTAU-22.1 affinity matured mutants for improved binding (Additional file 1: Table S2). Biotinylated tau peptides (Additional file 1: Table S2) were immobilized on Streptavidin (SA) Dip and Read biosensors for kinetics (ForteBio) at a concentration of 2.5 μg/ml. Real-time binding curves were measured by applying the sensor in ForteBio’s kinetics buffer containing 100 nM CBTAU mAbs for 600 s. To induce dissociation, the biosensor containing the mAb-tau peptide complex was immersed in ForteBio’s kinetic buffer without antibody for 600 s. The relative association and dissociation kinetic curves were compared to qualitatively assess the efficiency of CBTAU binding to peptides under different conditions. To assess the nature of interaction, binding profiles were determined using different ionic strengths buffers. To gauge specificity a set of phosphorylated tau peptides outside the epitope region were taken along (Additional file 1: Table S2).

### Binding affinities by octet biolayer interferometry

An indication for the improvement of the dmCBTAU-22.1 was obtained by measuring the affinity (K_D_) of both Fab-CBTAU-22.1 and Fab-dmCBTAU-22.1 using the Steady State Analysis. Fabs were used to get a 1:1 stoichiometry. Biotinylated tau peptide V1089–24 (Additional file 1: Table S2) at 1 μg/ml was immobilized on Streptavidin (SA) biosensors till a shift of ~ 1 nm is reached. Then it was dipped into ForteBio’s kinetics buffer containing a range of 13 Fab concentrations for each construct. This association step had a duration of 600 s for each concentration to reach equilibrium, followed by a 600 s dissociation step. The value of the equilibrium dissociation constant (K_D_) is obtained via steady state analysis by fitting a plot of response values, which is the average of the last 5 s of the association step, against the respective Fab concentrations.

### Affinity maturation

The coding sequence for scFv directed against CBTAU-22.1 epitope was cloned into an inducible prokaryotic expression vector containing the phage M13 pIII gene. Random mutations were deliberately introduced in the scFv by error prone PCR (Genemorph II EZClone Domain Mutagenesis kit) after which the DNA was transformed into TG1 bacteria. The transformants were grown to mid-log phase and infected with helper phages which provide all the genes required for phage assembly. ScFv expressing phages were rescued by a CT helper phage genome which lacks the infectivity domains N1 and N2 of protein pIII, rendering phage particles which are only infective if they display the scFv linked to the full length pIII [27]. Phage libraries were screened using magnetic beads coated with CBTAU-22.1 cognate peptide V1089–24 (Additional file 1: Table S2) in immunotubes. After three rounds of panning, individual phage clones were isolated and screened in phage ELISA for binding to V1089–24. Based on ELISA, clones were selected and converted into an IgG1 format to assess affinity in solution.

### Crystallization, data collection and structure determination of fab-CBTAU-22.1 in complex with peptide V1088–5 and fab-dmCBTAU-22.1 in complex with peptide V1088–23

For crystallization the Fab fragments (in 20 mM HEPES/NaOH pH 7.5, 7.55 mM NaCl) were incubated with 4 mM of the respective peptide on ice overnight and concentrated to a final concentration of about 50 mg/ml. The Fab:peptide complexes were subjected to a broad crystallization screening by mixing 0.1 μL protein solution and 0.1 μL reservoir with sitting drop vapor diffusion, varying also the protein concentration. Fab-CBTAU-22.1 with peptide V1088–5 (Additional file 1: Table S2) was crystallized from 0.20 M KCl, 0.10 M Hepes/NaOH pH = 7, 21.2% (*w*/*v*) PEG 5 K MME at a concentration of 25 mg/mL. Fab-dmCBTAU-22.1 with peptide V1088–23 (Additional file 1: Table S2) was crystallized from 10% (w/v) PEG8K, 0.10 M Tris/HCl pH = 7.0, 0.20 M MgCl_2_ at a concentration of 30 mg/mL. For cryo-protection crystals were briefly immersed in a cryo-solution consisting of 75% reservoir and 25% glycerol. X-ray diffraction data were collected at temperature of 100 K at the Swiss Light Source. Data were integrated, scaled and merged using XDS [25]. The structure was solved with MOLREP [48] and refined with REFMAC5 [49]. Manual model completion was carried out using Coot [18]. The quality of the final model was verified PROCHECK [28] and the validation tools available through Coot [18]. Data collection and refinement statistics for Fab-CBTAU-22.1 in complex with peptide V1088–5 and Fab-dmCBTAU-22.1 in complex with peptide V1088–23 are listed in Additional file 1: Tables S3 and S4.

### FRET based cellular immunodepletion assay

Homogenates were prepared from cryopreserved cortical grey matter of 17 sporadic AD patients acquired from the Newcastle Brain Tissue Resource biobank and post mortally assessed at Braak stages 5–6. Patients were all Caucasian between ages of 56 and 93 years old at time of death. Tissue was homogenized in homogenization buffer (10 mM Tris (Gibco), 150 mM NaCl (Gibco) containing protease inhibitors (cOmplete ULTRA tablets EDTA free, Roche) to obtain a 10% *w*/*v* pooled brain homogenate. The homogenate was centrifuged at 27.000×g, 10 min at 4 °C and supernatants of different patients were pooled and stored in aliquots at − 80 °C until used as seed in the immunodepletion assay. Individual antibody dilutions were prepared in PBS pH 7.4 (Sigma), mixed with brain extract in a 1:1 ratio in a 96 well PCR plate (Thermo Scientific), and incubated until the beads were washed. Protein-G DynaBeads (Life Technologies) were added in a 96-well PCR plate (Thermo Scientific) and washed twice with PBS, 0.01% Tween-20 (Sigma) by pulling down the beads with a magnet (Life Technologies). Wash buffer was removed completely and 10 μL of PBS, 0.1% Tween-20 were added to the beads together with 90 μL of the 1: 1 antibody-brain extract mixture. Samples were incubated over night at 4 °C, rotating at 5 rpm. The following day, the immunodepleted fractions were separated from the beads by pulling down the beads with the magnet, transferred to a new 96-well PCR plate and stored at − 80 °C until tested. Each condition was tested in duplicate. Immunodepleted fractions were incubated for 10 mins with Lipofectamine 2000 (Invitrogen) in Opti-MEM (Gibco) in a 96-well cell culture plate (Greiner Bio-one) before 5.5 × 103 HEK biosensor cells (provided by M. Diamond, Washington University School of Medicine) were added to each well. After a 2-day incubation at 37 °C, cells were washed twice with PBS, detached using Trypsin/EDTA (Gibco) and transferred to a polypropylene round bottom plate (Costar) containing FACS buffer (Hank’s Balanced Salt Solution (Sigma), 1 mM EDTA (Invitrogen), 1% FBS (Biowest)). Cells were then analyzed for FRET positivity by flow cytometry using a FACS Canto II (BD Bioscience). Each plate contained a brain extract only condition (to assess baseline FRET response) and an antibody isotype control. Results are reported as normalized values, relative to condition without antibody.

### Immunohistochemistry on post mortem human brain tissue

Post-mortem human brain tissues of asymptomatic (62–74 year old, Braak stage 1–2) and AD patients (62–81 years old, Braak stage 5) were obtained from the Netherlands Brain Bank (Amsterdam, The Netherlands). All donors, or their next of kin, had given informed consent for the use of brain tissue and clinical details for research purposes. Sections (5 μm-thick) from formalin-fixed paraffin embedded control and AD brain tissue (hippocampus) were mounted on coated glass slides (Menzel gläser superfrost plus, VWR international, Leuven, Belgium) and dried overnight at 37 °C. Slides were deparaffinized in xylene and rehydrated through descending alcohol concentrations. Endogenous peroxidase activity was blocked by incubating the slides 30 min in phosphate buffered saline (PBS; pH 7.4) containing 0.3% H_2_O_2_. Subsequently, sections were subjected to pre-treatment: slides were heated in 10 mM citrate buffer (pH 6.0) in the autoclave. After allowing the heated sections to re-equilibrate to room temperature (RT), sections were rinsed in PBS. CBTAU-22.1 and anti-phosphorylated Tau (Ser^202^/Thr^205^; clone AT8, Thermo Fisher) were diluted in antibody diluent (Immunologic, Duiven, The Netherlands) and incubated overnight at RT. After incubation, sections were thoroughly rinsed in PBS and incubated with ready-to-use goat-anti-mouse/rabbit EnVision+HRP (Dako, Glostrup, Denmark) for 30 min at RT, followed by rinsing in PBS. To visualise the staining 3,3′-diaminobenzidine (DAB; Dako) was used. Slides were counterstained with haematoxylin, dehydrated and mounted with Quick D mounting medium (Klinipath, Duiven, The Netherlands).

### Production recombinant tau fragments (tau1–415-Mxe-CBD) for ligation

DNA inserts for tau fragments with suitable overlaps to the pTXB1 vector (New England Biolabs) were obtained commercially (IDT) and cloned into the expressed protein ligation vector using the IMPACT kit and associated protocols (New England Biolabs). After cloning, the plasmid was amplified in *E. coli* NEB5α cells (New England Biolabs). Transformation of *E. coli* BL11 cells (New England Biolabs) was performed by mixing 25 μL cell suspension and 2 μL plasmid and incubating on ice for 30 mins. The mixture was heated to 42 °C for 30 s after which 475 μL LB medium was added followed by incubation at 37 °C for 1 h. 100 μL was spread on LB-Ampicilin+ agar plates and incubated overnight at 37 °C. 4 colonies were picked and transferred to 4 mL LB-Ampicilin+ and incubated for 8 h at 37 °C after which the mixture was diluted to 50 mL and further incubated overnight. 8 × 500 mL LB-Ampicilin+ was inoculated with sufficient overnight culture to reach an OD500 of 0.2 and incubated at 37 °C for approximately 2 h until the OD280 had reached 0.6. IPTG (Sigma) was added to a final concentration of 1 mM followed by further incubation for 3 h. Cells were harvested by centrifugation and stored at − 80 °C as 8 pellets until further use.

### Expressed protein ligation

A pellet containing tau (1–415)-Mxe-CBD was added to 20 mL of Bugbuster MasterMix (Millipore) supplemented with a Pierce cOmplete protease inhibitor tablet (Thermo Scientific) and incubated for 30 mins at RT. After centrifugation for 10 min, the 5 mL lysate was added in its entirety to 3 mL of chitin resin beads (New England Biolabs) and incubated for 2 h at RT. The resin was then added to 10 mL of PBS supplemented with 0.02% Tween-20 (Merck), 50 mM MESNa (Sigma) and 1 mM TCEP (Sigma) as well as 2 mg of phosphorylated tau peptide CL22D-P (CIDMVD(pS)PQLATLADEVSASLAKQGLEPEA, Pepscan, The Netherlands). The ligation mixture was incubated for 48 h at RT after which the resin was decanted, the supernatant concentrated on an Amicon 10 k filter (Sigma) and buffer exchanged to 3.5 mL PBS on a PD10 column (Thermo Scientific). The crude ligate was run by gravity assist over a bed of 2 mL CaptureSelect C-tag beads (Thermo Scientific), followed by washing with 20 mL PBST (PBS with 0.02% Tween-20) and elution with 10 mL 100 mM pH 3.5 citrate buffer. The eluate was concentrated and buffer exchanged on a PD10 column as above. The purified ligation product in PBS (typically 1 mg/mL) was stored at − 20 °C until use. SEC-MALS characterization was performed as described above for the other recombinant proteins. Western blot analysis was performed by running 2N4R-tau and pS422-tau on a 4–12% BisTris gel (Novex) at 200 Vc in MOPS buffer followed by transfer onto a PVDF membrane (Invitrogen) using iBlot equipment. Primary antibody concentrations was 5 μg/mL and a dilution of 1:20000 was employed for the HRP-goat-anti-human conjugate antibody (Jackson). The blot was stained with the FemtoWest kit (Pierce) and imaged on a Biorad ChemiDoc XRS.

### In vitro tau aggregation assay

Stock solutions of 500 μM thioflavin T (ThT) (Sigma-Aldrich, St Louis, MO, USA) and 55 μM heparin (Mw = 17–19 kDa; Sigma-Aldrich, St Louis, MO, USA) were prepared by dissolving the dry powders in reaction buffer (0.5 mM TCEP in PBS, pH 6.7), and filtered through a sterile 0.22 μm pore size PES membrane filter (Corning, NY, USA) or a sterile 0.22 μm pore size PVDF membrane filter (Merck Millipore, Tullagreen, Cork, IRL) respectively. The concentration of the ThT solution was determined by measurement of the absorption at 411 nm (extinction coefficient 22,000 M^− 1^ cm^− 1^). Concentration of pS422-tau was determined through its absorption at 280 nm (extinction coefficient 0.31 ml mg^− 1^ cm^− 1^). For spontaneous conversions, mixtures of 6.5 μM pS422-Tau in 200 μl reaction buffer containing 7 μM heparin and 50 μM ThT were dispensed into 96-well plates (Thermo Scientific, Vantaa, Finland) that were subsequently sealed with plate sealers (R&D Systems, Minneapolis, MN). To assess the effect of IgG on the conversion, pS422-tau and IgG were mixed and incubated for 20 min in reaction buffer before the addition of heparin and ThT. Kinetic measurements were monitored at 37 °C in a Biotek Synergy Neo2 Multi-Mode Microplate Reader (Biotek, VT, USA) by measuring ThT fluorescence at 485 nm (20 nm bandwidth) upon excitation at 440 nm (20 nm bandwidth) under continuous shaking (425 cpm, 3 mm).

### Atomic force microscopy

For each sample, 20 μl of pS422-tau was deposited onto freshly cleaved mica surface. After 3 min incubation, the surface was washed with double-distilled water and dried with air. Samples were imaged using the Scanasyst-air protocol using a MultiMode 8-HR and Scanasyst-air silicon cantilevers (Bruker Corporation, Santa Barbara, USA). Height images of 1024 × 1024 pixels in size and surface areas of 10 × 10 μm were acquired under ambient environmental conditions with peak force frequency of 2 KHz.

### Preparation of post-mortem human AD brain-derived PHFs for co-injection in transgenic P301L mice

*Post-mortem* tissue from the parietal cortex obtained from 5 histologically confirmed Alzheimer patients was partially purified by a modified method from [20]. Briefly, 5 g of frontal cortex was homogenized in 10 volumes of cold buffer H (10 mM Tris, 800 mM NaCl, 1 mM EGTA and 10% sucrose, pH 7.4) using a glass/Teflon Potter tissue homogenizer (IKA Works, Inc.; Staufen, Germany) at 1000 rpm. The homogenized material was centrifuged at 27,000 x g for 20 min. The pellet was discarded and the supernatant was adjusted to a final concentration of 1% (*w*/*v*) *N*-lauroylsarcosine and incubated for 2 h at 37 °C. Subsequently the supernatant was centrifuged at 184,000 x g for 90 min at 20 °C. The pellet was carefully washed in PBS and resuspended in 750 μL PBS, aliquoted and frozen at − 80 °C. The quality of the PHF-tau preparations was evaluated by AT8/AT8 phospho-aggregate-selective MSD and by Western blotting (Additional file 1: Figure S4) using pan tau antibody hTau10 and anti phospho-tau antibody AT8 (pS^202^/pT^205^/pS^208^) [30]. A range of 2.5–20 μl of 1: 10 diluted PHF were resolved on SDS-PAGE (4–12% Bis–Tris Novex NuPAGE gel; Invitrogen) and subsequently transferred onto a nitrocellulose membrane. The membrane was blocked overnight in 1X TBS-T with 5% non-fat dry milk (blocking buffer). In house HRPO-labelled AT8 and hTau10 were used at 1 μg/ml in 2.5% BSA in TBS-T and incubated for 2 h at room temperature. The membranes were then washed three times for 5 min each in TBS-T. The membrane was washed three times for 5 min and developed using the Supersignal West Pico kit (Pierce). Images were obtained on the ImageQuant LAS-4000 (GE Healthcare).

### Animals and stereotactic injections

Transgenic Tau-P301L mice, expressing the longest human tau isoform with the P301L mutation (tau-4R/2 N-P301L) [46] were injected at the age of 3 months. Two groups of 15 mice received unilateral hippocampal injection of 1 pmol AD-derived PHFs (3 μl) in an equimolar mixture with dmCBTAU-22.1 or anti-rabies IgG2a isotype control antibody, respectively. The concentration of PHFs is expressed in mols of monomeric Tau and was determined by western blotting using recombinant tau protein as reference. Stereotactic injections in the right hippocampus (TILT-corrected point of injection; CA1, AP -2.0, ML + 1.6 (from bregma), DV 1.4 mm (from dura)) were performed with a semi-automatic stereodrive drill-injection robot (Neurostar) according to the following settings: Hamilton 10 μl syringe, 30G needle point type 4 (45° beveled, not curved), opening 30–45° angle outwards at an injection rate 0.20 μl/min). All experiments were performed in compliance with protocols approved by the local ethical committee and national institutions adhering to AAALAC guidelines.

### PHF extraction from mouse brain

Two months after injection, tissue from the *ipsilateral* hemisphere was weighed and homogenized in 6 volumes of buffer H (10 mM Tris, 800 mM NaCl, 1 mM EGTA and 10% sucrose, pH 7.4) using a FastPrep-24 5G benchtop homogenizer (MP Biomedicals).

*Biochemical analysis MesoScale Discovery (MSD).*

Coating antibody (AT8) was diluted in PBS (1 μg/mL) and aliquoted into MSD plates (30 μL per well) (L15XA, MSD, Rockville, MD, USA), which were incubated overnight at 4 °C. After washing with 5 × 200 μL of PBS/0.5%Tween-20, the plates were blocked with 0.1% casein in PBS and washed again with 5 × 200 μl of PBS/0.5% Tween-20. After adding samples and standards (both diluted in 0.1% casein in PBS), the plates were incubated overnight at 4 °C. Subsequently, the plates were washed with 5 × 200 μL of PBS/0.5% Tween-20, and SULFO-TAG™ conjugated detection antibody (AT8) in 0.1% casein in PBS was added and incubated for 2 h at room temperature while shaking at 600 rpm. After a final wash (5 × 200 μL of PBS/0.5%Tween-20), 150 μL of 2 x buffer T (MSD) was added, and plates were read with an MSD imager. Raw signals were normalized against a standard curve consisting of 16 dilutions of a sarcosyl-insoluble prep from *post-mortem* AD brain (PHF) and were expressed as arbitrary unit (AU) PHFs. Statistical analysis (ANOVA with Bonferroni post test) was performed with the GraphPad prism software and with an ‘in house’ developed application for automated analysis.

### Data deposition

The atomic coordinates and structure factors of the co-crystal structures of CBTAU-22.1 Fab in complex with tau peptide V1088–5 and dmCBTAU-22.1 Fab in complex with tau peptide V1088–23 are being deposited in the Protein Data Bank, www.rcsb.org (PDB ID codes 6H06 and 6H0E) and will be released immediately upon publication.

## Results

**Fig. 1:**
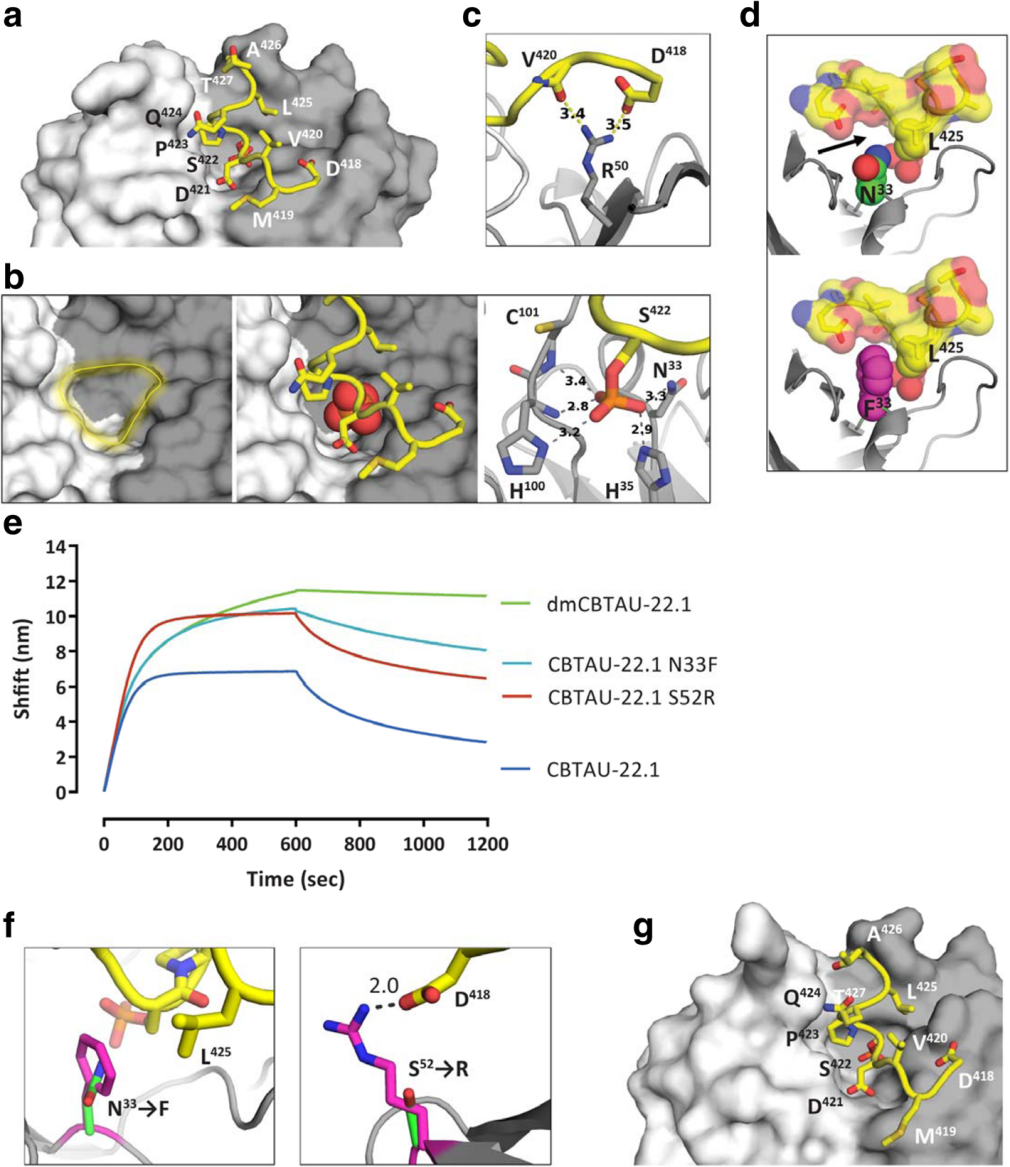
Affinity maturation of CBTAU-22.1. **a** Co-crystal structure of Fab CBTAU-22.1 with tau peptide V1088–5. The Fab’s molecular surface is plotted with heavy chain in grey and light chain in white. Tau peptide is plotted in yellow. **b** Peptide binding is driven by the Ser^422^ phosphate hotspot. Its binding pocket (left panel) is formed in the groove between light and heavy chains. The phosphate (plotted here as spheres) is buried deeply in the pocket and fully disolvated (central panel). Multiple hydrogen bonds are formed to bind the phosphate hotspot in the pocket (right panel). **c** Arg^50^ buried by peptide binding, second example of charge-charge interaction between CBTAU-22.1 and Tau. **d** Design of the Asn^33^ → Phe mutant based on the structure of the wild type CBTAU-22.1. Heavy chain Asn^33^ (green) interaction with tau is water mediated. Water cavity surrounding the residue in the co-crystal structure is indicated with black arrow in the upper panel. Phenylalanine (magenta, lower panel) has been identified as a mutation with high shape complementarity with the pocket, forming hydrophobic interactions with Leu^425^. **e** Association and dissociation profiles for the parental antibody, variant antibodies with either the Ser^52^ → Arg mutation derived by random mutagenesis or the rationally-designed Asn^33^ → Phe, and a variant with both mutations combined (dmCBTAU-22.1) to peptide V1089–24 as determined by Octet biolayer interferometry. **f** Interactions introduced by the heavy chain mutations confirmed by the co-crystal structure of the double mutant. Wild type residues are plotted in green, and the mutations in magenta. Tau peptide amino-acids are plotted in yellow, and the antibody heavy chain in grey Left panel; Asn^33^ (green) mutated to Phenylalanine (magenta) resulted in formation of hydrophobic contacts with tau’s Leu^425^. Right panel: Ser^52^ (green) was mutated to Arginine (magenta) and resulted in addition of a charge-charge interaction with tau’s Asp^418^. **g** Co-crystal structure of Fab dmCBTAU-22.1 with tau peptide V1088–23. The Fabs’ molecular surface is plotted with heavy chain in grey and light chain in white. Tau peptide is plotted in yellow. Tau peptide sequences are listed in Additional file 1: Table S2

To improve the affinity of CBTAU-22.1, we employed a combination of random mutagenesis and rational design approaches. The random error prone PCR approach led to the identification of several variants with improved affinity to peptides encompassing the epitope of CBTAU-22.1 (Additional file 1: Table S1), the best of which being a variant containing a Ser^52^ → Arg mutation in the CDR2 of the heavy chain. The rational design was based on a co-crystal structure of Fab CBTAU-22.1 with tau peptide (Fig. 1a), which revealed polar interaction with the Ser^422^ phosphate playing a pivotal role in the hotspot from the center of the complex. The phosphate, together with 4 water molecules, gets buried in the cavity formed in the groove between the heavy and the light chains (Fig. 1b). In addition to the bridging waters, it forms hydrogen bonds with the heavy side-chains His^35^, His^100^, Asn^33^ and the backbone amide nitrogen of Cys^101^. Another charge buried upon binding of CBTAU-22.1 to tau is the guanidine group of Arg^50^ from the heavy chain (Fig. 1c). Looking for the opportunities to design mutants with better affinities, we focused on forming new hydrophobic interactions and identified Asn^33^ as one possible site for mutation (Fig. 1d). Asn^33^ forms a hydrogen bond with the phosphate, but does not efficiently interact with the rest of the tau peptide. Most interactions are mediated by waters filling the small cavity surrounding the sidechain of the residue. We have scanned through all possible mutations and hypothesized that the phenylalanine side chain would fill the entire pocket, expel the unstable water molecules and form hydrophobic contacts with Leu^425^. Our assumption was that even though the hydrogen bond with the phosphate would be lost, a Asn^33^ → Phe mutation would result in net gain in binding free energy due to multiple unfavorable water molecules being expelled and additional interactions being formed. While the Ser^52^ → Arg and Asn^33^ → Phe mutations each enhanced binding, the combination of these two mutations led to a double mutant of CBTAU-22.1 (from here on referred to as dmCBTAU-22.1) with significantly improved binding affinity, emphasized especially by its strikingly slower dissociation profile (Fig. 1e). Affinity measurements showed that the binding of dmCBTAU-22.1 (Kd = 240 ± 35 nM) to tau peptide is improved by a factor of ~ 25 relative to parental CBTAU-22.1 (Kd = 5.6 ± 0.6 μM) (Additional file 1: Figure S1). In addition, the binding profiles of Fab fragments suggested that the much higher affinity of dmCBTAU-22.1 relative to the parental antibody is due to a much slower dissociation rate without significant effect on association kinetics. A co-crystal structure of the Fab of dmCBTAU-22.1 with tau peptide confirms the predicted phenylalanine conformation (Fig. 1f, left panel) and shows that the Ser^52^ → Arg mutation resulting from the random mutagenesis approach gives rise to a charge-charge interaction with Asp^418^, explaining the improved binding affinity (Fig. 1f, right panel). Nevertheless, the double mutant reveals the same overall binding mode as the wild-type antibody (compare Fig. 1, panels a and g).

We have previously shown that the interaction between CBTAU-22.1 and tau is affected by ionic strength [35]. A similar dependency was observed for dmCBTAU-22.1 (Additional file 1: Figure S2) indicating that the maturation process did not alter the epitope binding mode. Furthermore, we observed that in addition to the intrinsic optimization of affinity, avidity plays an important role in binding of the antibodies to tau, resulting in a very slow dissociation of dmCBTAU-22.1. Further binding experiments employing a set of different phosphorylated tau peptides (Additional file 1: Table S2) confirmed also that the specificity of the parental antibody was retained (Additional file 1: Figure S3).

**Fig. 2:**
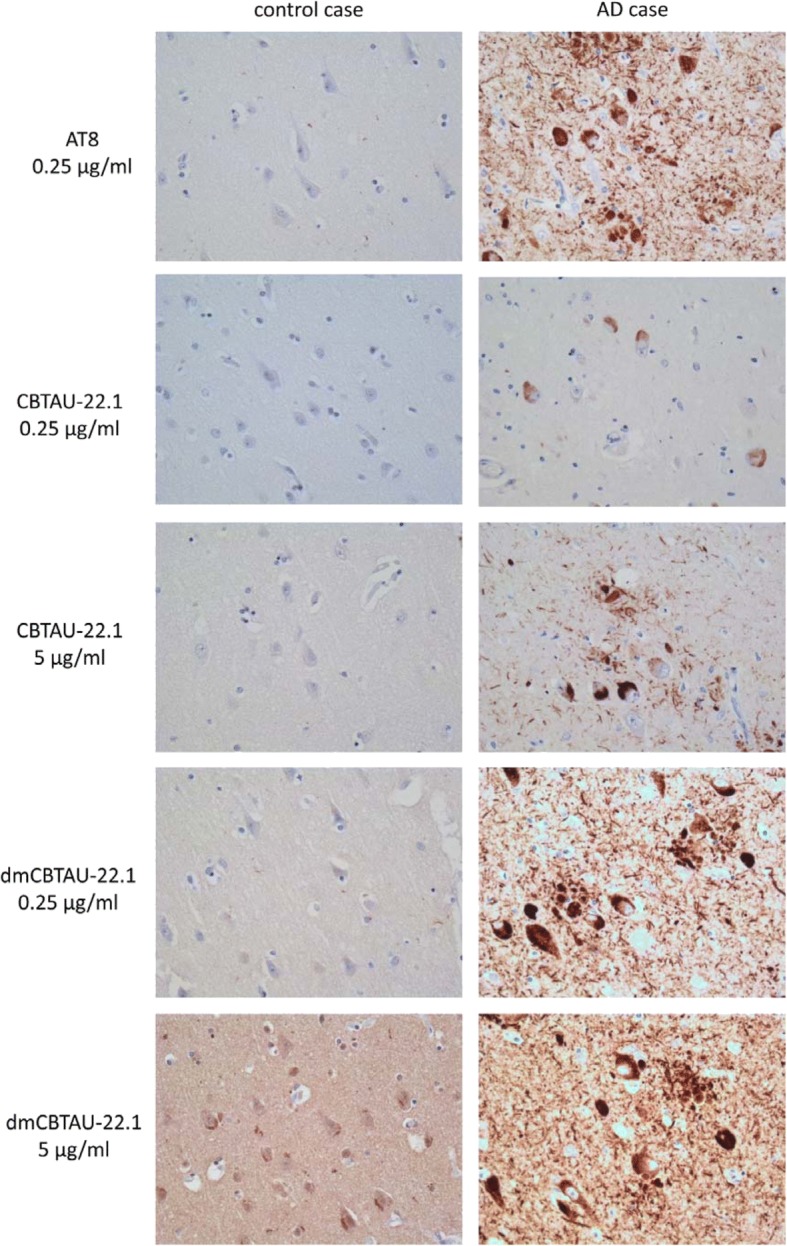
Immunohistochemical detection of pathological tau in AD brain tissue. Immunohistochemistry with CBTAU-22.1 and dmCBTAU-22.1 on non-demented control and AD human brain tissue (hippocampus) was performed using two different concentrations of antibody: 0.25 μg/mL and 5 μg/mL. AT8 immunostaining (0.25 μg/mL) was applied on an adjacent section for comparison

The ability of the affinity improved dmCBTAU-22.1 to specifically recognize pathogenic tau structures in AD brain was compared to that of parental antibody (Fig. 2). In post mortem AD brain tissue, CBTAU-22.1 (5 μg/mL) detects pathological tau structures, which include (pre)tangles, neurofibrillary threads, and neuritic plaques. No immunoreactivity was observed in nondemented control brain tissue. While AT8 shows strong immunoreactivity of pathological tau structures at a concentration of 0.25 μg/mL, parental CBTAU-22.1 showed weak immunoreactive staining of tangles at this concentration. The detection of pathological tau was strongly improved with dmCBTAU-22.1 which showed intense staining of pathological tau at 0.25 μg/mL. These results further confirm the significant improvement in affinity and preservation of specificity of dmCBTAU-22.1.

**Fig. 3:**
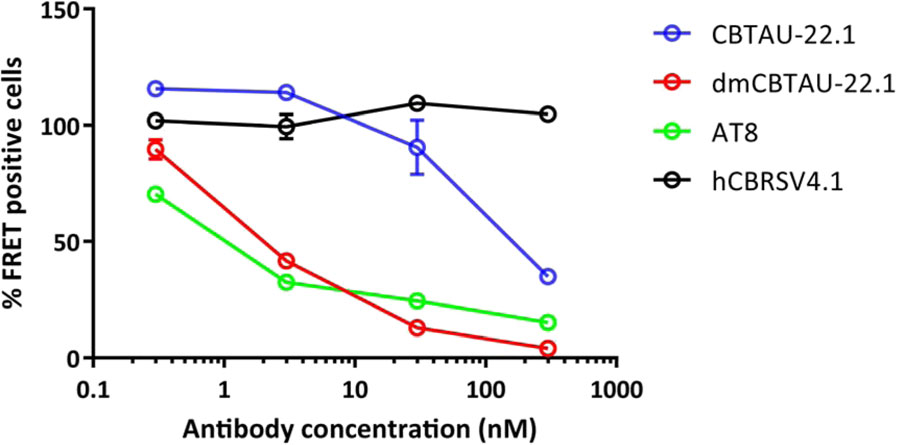
Enhanced immunodepletion of AD seeds by affinity improved dmCBTAU-22.1. Residual seeding activity of human AD brain homogenates following immunodepletion with different concentrations of CBTAU-22.1 (blue) and dmCBTAU-22.1 (red) as measured by FRET signal in biosensor cells expressing the microtubule repeat domains of tau (aa 243–375) fused either to yellow or cyan fluorescent protein. Uptake of exogenous tau aggregates into the cells results in aggregation of the tau fusion proteins, which is detected by FRET. As positive and negative controls, a human IgG1 chimeric version of murine anti-PHF antibody AT8 (green) and anti-RSV-G antibody CBRSV-4.1 (black) were taken along, respectively. Error bars indicate the SD of two independent experiments

We next assessed the effect of affinity improvement on the ability of the antibodies to bind PHFs and thus potentially block the propagation and spreading of tau pathology. To this end, the antibodies are incubated with AD brain homogenate in an immunodepletion step and subsequently residual seeding capacity is measured in a FRET-based assay [24]. As Fig. 3 shows, both CBTAU-22.1 and dmCBTAU-22.1 are capable of depleting PHFs from AD brain homogenate in a concentration dependent manner. As expected, dmCBTAU-22.1 was significantly more potent in this assay than parental CBTAU-22.1, with virtually complete seeding inhibition at he highest antibody concentration tested. Negative control antibody RSV-4.1 which does not bind tau was indeed unable to diminish PHF seeding efficiency even at the highest concentration.

**Fig. 4:**
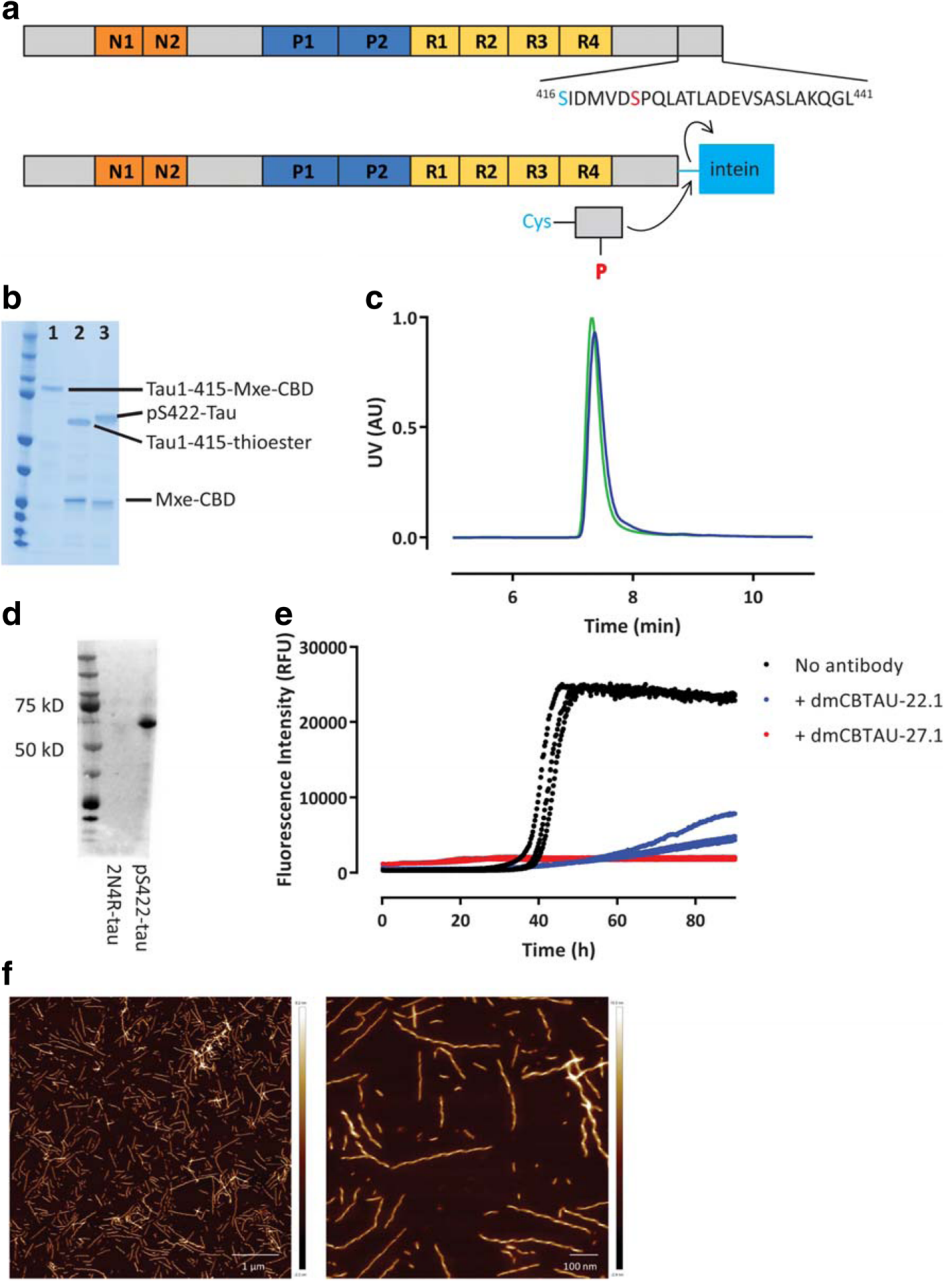
Inhibition of in-vitro aggregation of pS422-tau by dmCBTAU-22.1. **a** Schematic representation of the chemical ligation process applied to preparation pS422-tau. Ser^422^ (red) is phosphorylated in the CBTAU-22.1 epitope and Ser^416^ (blue) is mutated into Cys as a result of the chemical ligation process. **b** SDS-PAGE analysis of the ligation reaction progress. Lane 1 corresponds to the full tau1–415-Mxe-CBD multidomain protein produced in *E. coli* after cloning into the pTXB1 vector. For the purposes of this analysis the protein was purified via His-tag affinity chromatography. Lane 2 corresponds to the thioester product obtained by treatment of the Lane 1 product with excess MESNa. Lane 3 represents the ligation product obtained by reaction of the Lane 2 product with tau peptide CL22D-P. **c** Size Exclusion Chromatography profiles of pS422-tau (green) and non-phosphorylated 2N4R-tau (blue). **d** Western blot of pS422-tau and non-phosphorylated 2N4R-tau with dmCBTAU-22.1. **e** Aggregation of pS422-tau in the absence (black) or presence of dmCBTAU-27.1 (red) or dmCBTAU-22.1 (blue) as monitored continuously by ThT fluorescence. The molar ratio between pS422-tau and IgG was 1: 0.6 in both cases. Each experimental condition was tested in three independent replicates, the red triplicates and two of the blue triplicates overlap and cannot be visually distinguished. **f** Atomic force microscopy of pS422-tau fibrils. The two panels correspond to AFM images with sizes of 6 × 6 μm and 1.5 × 1.5 μm, respectively

Since the epitope recognized by CBTAU-22.1 is in the proximity of the MTBR which forms the core of PHF aggregates and is known to be crucial for the initial tau nucleation, we assessed the ability of the antibody to interfere with early stages in the tau aggregation process. We have previously reported on a highly reproducible rTau aggregation assay displaying all NDP features that can be used to assess the ability of antibodies to interfere with the aggregation of tau [1]. However, since CBTAU-22.1 recognizes an epitope phosphorylated at Ser^422^ which is not present on recombinantly produced tau we employed here a native protein ligation [33] strategy where a large recombinantly produced tau^1–415^ protein fragment is coupled to a small, synthetic peptide encompassing tau^416–441^ quantitatively phosphorylated at Ser^422^ (Fig. 4a). We employed a previously described ligation protocol for phosphorylated tau species [9, 37, 38], but modified it by selecting a different ligation site (as discussed in the Materials and Methods) and by adding an additional C-terminal purification tag. Progress of the ligation was monitored by capturing intermediates and visualizing on SDS-PAGE (Fig. 4b), and purification of the ligated product was performed by removal of the excess peptide and additives based on their low molecular weight followed by purification through the Ctag. Native size analysis of pS422-tau indicated that the ligated material was homogeneous, monomeric and had same molecular weight as 2N4R-tau (Fig. 4c). In addition, a Western Blot with dmCBTAU-22.1 confirmed that the phosphorylation at Ser^422^ is present in the pS422-tau ligation product (Fig. 4d).

Next, we investigated the behavior of pS422-tau in the in vitro aggregation assay. The aggregation kinetics profiles in Fig. 4e showed that, similar to 2N4R, the aggregation of pS422-tau shows the expected features of an NDP process with well-defined nucleation (nuclei formation) followed by an exponential fibril growth step [1]. AFM imaging further confirmed the formation of a homogeneous population of twisted fibrils similar in appearance to those observed for the aggregation of non-phosphorylated tau (Fig. 4f).

We have previously described an antibody, dmCBTAU-27.1, targeting the tau PHF motif located in the MTBR which is capable of completely blocking the aggregation process for non-phosphorylated recombinant tau [1]. Since the epitope of this antibody is distant from the C-terminus, its inhibiting ability should not be affected by phosphorylation introduced at Ser^422^. Indeed, dmCBTAU-27.1 blocked the aggregation of pS422-tau at similar antibody concentrations as used before with non-phosphorylated tau (Fig. 4e, red traces). Upon incubation with dmCBTAU-22.1 at similar concentration we could clearly observe inhibition of the tau aggregation process (Fig. 4e, blue traces) even though the effect was not as strong as for dmCBTAU-27.1. This is most probably due to the epitope of CBTAU-22.1 being located outside of the key tau aggregation region as opposed to dmCBTAU-27.1 and is therefore arguably less well situated for blocking aggregation. Nonetheless, the observed behavior suggests that, in addition to a major inhibitory effect of PHF spreading, dmCBTAU-22.1 could also interfere with earlier stages of tau pathogenesis which broadens its scope both in prevention as well as therapeutic intervention.

**Fig. 5:**
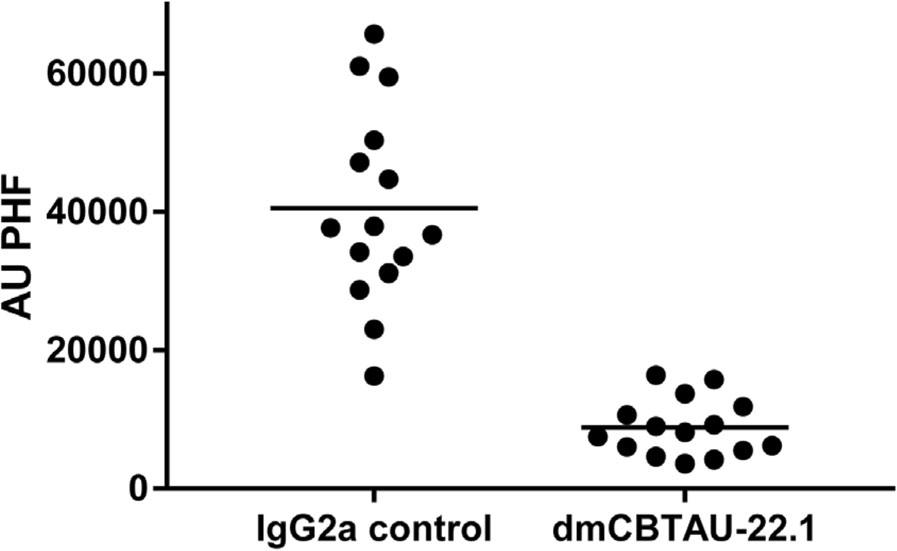
Inhibition of PHF spreading by dmCBTAU-22.1 in a co-injection experiment using transgenic P301L mice. Two groups of 15 mice received unilateral (right hemisphere) hippocampal injection of 1 pmol AD-brain derived PHFs (concentration expressed in mols of monomeric tau) in an equimolar mixture with dmCBTAU-22.1 or an anti-rabies IgG2a isotype control antibody. Shown is the amount of PHF pathology, as determined by biochemical analysis (AT8/AT8 MSD) and displayed in arbitrary units (AU), in total P301L brain homogenates of the injected brain hemisphere two months after injection

To evaluate whether the in vitro functionality of dmCBTAU-22.1 translates to in vivo activity, we co-injected human AD-derived PHFs with equimolar amounts of dmCBTAU-22.1 expressed as mouse IgG2a in P301L mice following a protocol previously described for synthetic K18 seeds [36]. In this approach PHF-tau seeds derived from human AD brain are stereotactically injected in the hippocampal regions of P301L transgenic mice at the age of 3 months at which cell autonomous aggregation has not started. In the absence of antibody, PHF-tau seeds induce tauopathy in the injected hemisphere and to a lesser degree in the connected contralateral region. Biochemical analysis (AT8/AT8 MSD on total homogenates of the injected hemisphere) (Fig. 5) showed a 78% reduction in the amount of PHFs by the CBTAU-22.1 antibody (*P* < 0.0001) in comparison to the mouse anti-rabies IgG2a control group.

## Discussion

Tau pathogenesis is a complex process characterized by a series of conformational changes, aggregation and a cascade of phosphorylation events. In the absence of reliable in vivo models there are numerous controversies regarding the most efficient strategies to interfere and prevent tau pathogenesis by passive immunotherapy. However, regardless of the targeted mechanism, both the choice of tau epitope and the strength with which these are bound are expected to be pivotal. Antibody CBTAU-22.1 was, together with 51 other anti-tau antibodies, originally isolated from peripheral IgG^+^ memory B cells from asymptomatic donors [35]. We have previously reported that CBTAU-22.1 recognizes a Ser^422^ phosphorylated C-terminal epitope which has been associated with AD [4, 10, 12, 23, 41]. Furthermore, CBTAU-22.1 was shown to specifically recognize pathological tau deposits in post-mortem brain tissue and to have inhibitory activity in an in vitro tau aggregation assay using PHFs derived from P301S mice, suggesting a therapeutic potential of this antibody. However, presumably due to its modest affinity for tau, this activity was low (e.g. compared to that of murine anti-PHF antibody AT8) which would likely limit its therapeutic application. We used a combination of random mutagenesis and structure-based design to generate a mutant antibody with increased affinity. Based on its apo structure (PDB 5V7U), we predicted that the Ser^422^ phosphate plays the major role in the hotspot interaction between the antibody and tau, with hydrogen bonds with heavy chain His^35^, His^100^, Asn^33^ and the backbone amide nitrogen of Cys^101^ as visible in the apo structure through the binding of a buffer phosphate molecule [35]. This hypothesis is confirmed here by the co-crystal structure of Fab CBTAU-22.1 with tau peptide which guided us in deriving the Asn33 → Phe mutation. By combining this mutation with a Ser^52^ → Arg that was identified by random mutagenesis, we generated a significantly improved antibody, dmCBTAU-22.1 that has the same binding mode as CBTAU-22.1 in all measured parameters.

In post mortem brain tissue, dmCBTAU-22.1 specifically stains pathological tau structures with similar intensities to well-known PHF antibody AT8. This affinity for pathological tau aggregates translates into a significantly increased ability to deplete and neutralize PHFs from AD brain lysates that again is comparable in efficiency to AT8. While CBTAU-22.1 reduced PHF seeding efficiency to 35% at its highest concentration tested, dmCBTAU-22.1 achieved a similar effect at a 100 times lower concentration and completely depleted the PHF seeding at the highest concentration tested. These results confirm that increased affinity leads to increased potency. This would translate into lower required drug dose and thus alleviate the difficulty of passing sufficient amounts of antibody across the blood brain barrier.

To assess the potential ability of dmCBTAU-22.1 to interfere with the aggregation of tau, we used chemical ligation to prepare homogeneous tau with phosphorylation at Ser^422^. This strategy combines the advantage of peptide chemistry, the ability to introduce modified amino acids in a fully controlled way, with the advantage of recombinant expression, the ability to produce long sequences. In contrast to other conjugation methodologies, this approach is traceless: it requires no added linkers and affords a natural backbone. Selection of a suitable ligation site is key since the chemistry behind it demands the presence of a cysteine residue. One can: (1) take advantage of a cysteine residue already present, (2) employ the cysteine as a relatively close mimic of a serine residue or (3) chemically transform the cysteine into an alanine residue. Approach (1) was not available since there is no cysteine near the CBTAU-22.1 epitope. We decided on approach (2) since it leaves the possibility to maintain the two cysteine residues in tau, the oxidation state of which has an impact on aggregation; in contrast approach (3) would necessarily mutate these to alanines. We did not find in any of our studies any detrimental effect attributable to the resulting S416C mutation which we controlled for by preparing and testing ligated material from the corresponding non-phosphorylated peptide part. Using this tailormade pS422-tau protein in the in vitro aggregation assay, we have shown that dmCBTAU-22.1 not only binds with high affinity to pathological tau, it also interferes with the tau aggregation process that underpins pathology formation. Interestingly, this represents the first time we observed such inhibition for an antibody binding outside the core aggregation domain. These two properties, neutralization of pathological tau species and ability to interfere with aggregation, translate into significant functionality in P301L transgenic mice. Immunotherapy against tau represents a promising strategy for treating AD and other tauopathies considering the direct link and strong association between tau pathology and loss of cognition [8, 34, 43]. Multiple studies in mice suggest that several anti-PHF antibodies of non-human origin may have therapeutic potential (reviewed in [42]). The only other antibody against pS422 that has been structurally characterized is rabbit antibody Rb86 (PDB 5DMG) [11]. While comparison of the structures reveals differences in the mode of binding of the two antibodies, i.e. binding of CBTAU-22.1 is centered around the buried phosphate pS422 while that of Rb86 is strongly driven by hydrophobic interactions, the demonstrated efficacy of a murine IgG1 version of Rb86 (MAb86) in reducing tau pathology in a mouse model of AD [14] provides further support for the potential of targeting the pS422 epitope. The data presented here in combination with the fact that dmCBTAU-22.1 is a fully human antibody, makes this antibody a particularly promising candidate for therapeutic intervention. As mentioned, the most efficient strategies to interfere and prevent tau pathogenesis by passive immunotherapy is subject of debate. For example, Congdon et al., [15] have shown that binding to soluble phospho tau, rather than to aggregated PHFs, correlated with both intro- and extracellular antibody-mediated clearance of phospho tau in primary neurons, and improvement in cognition in the htau mouse model. These findings suggest that, next to tau spreading and aggregation, prevention of neurotoxicity may be important for effective immunotherapy. Assessment of the ability of dmCBTAU-22.1 to prevent additional aspects of tau pathogenesis, like neurotoxicity and actual neurodegeneration, will be subject of future studies.

## Additional file

Additional file 1: **Table S1.** Overview of mutations investigated during the maturation process. **Table S2.** Names and sequences of tau peptides used in this study. **Table S3**. Data collection for Fab-CBTAU-22.1 in complex with peptide V1088-5 and Fab-dmCBTAU-22.1 in complex with peptide V1088-23. **Table S4.** Refinement statistics for Fab-CBTAU-22.1 in complex with peptide V1088-5 and Fab-dmCBTAU-22.1 in complex with peptide V1088-23. **Figure S1.** Binding affinities of the antigen binding domains of CBTAU-22.1 (left) and dmCBTAU-22.1 (right) to biotinylated peptide V1089-24 as determined by Octet Biolayer Interferometry. **Figure S2.** Association and dissociation kinetics for the binding of CBTAU-22.1 (left) and dmCBTAU-22.1 (right) to peptide V1089-24 at different ionic strengths. **Figure S3.** Association and dissociation kinetics for the binding of CBTAU-22.1 (left) and dmCBTAU-22.1 (right) to a panel of phosphorylated tau-derived peptides**.** Neither of the antibodies shows unspecific binding to tau peptides that do not contain the pSer^422^ epitope. **Figure S4.** Western blot detection of PHFs using anti-tau antibodies hTau10 (left) and AT8 (right). (DOCX 2093 kb)
