# Supplementary material for: Enhancement of therapeutic potential of a naturally occurring human antibody targeting a phosphorylated Ser422 containing epitope on pathological tau
Source: Acta Neuropathol Commun. 2018 Jul 12;6:59. doi: 10.1186/s40478-018-0562-9 (PMC6042391; doi:10.1186/s40478-018-0562-9)

**Additional files**

Enhancement of therapeutic potential of a naturally occurring human antibody targeting a phosphorylated Ser^422^ containing epitope on pathological tau

**Jeroen van Ameijde^1#^, Rosa Crespo^1#^, Roosmarijn Janson^1#^, Jarek Juraszek^1#^, Berdien Siregar^1^, Hanneke Verveen^1^, Imke Sprengers^1^, Tariq Nahar^1^, Jeroen J. Hoozemans^2^, Stefan Steinbacher^3^, Roland Willems^4^, Lore Delbroek^4^,** **Marianne Borgers^4^, Koen Dockx^5^, Kristof Van Kolen^4^, Marc Mercken^4^, Gabriel Pascual^6^, Wouter Koudstaal^1^, Adrian Apetri^1*^**

^1^Janssen Prevention Center, Janssen Pharmaceutical Companies of Johnson & Johnson, Archimedesweg 6, 2333 CN, Leiden, the Netherlands; ^2^Department of Pathology, Amsterdam Neuroscience, VU University Medical Center, De Boelelaan 1117, 1081 HV, Amsterdam, the Netherlands; ^3^Proteros Biostructures GmbH, Bunsenstraße 7a, 82152 Planegg, Germany; ^4^Janssen Neuroscience Discovery, Janssen Pharmaceutical Companies of Johnson & Johnson, Turnhoutseweg 30, 2340 Beerse, Belgium; ^5^Molecular and Cellular Pharmacology, Discovery Sciences, Janssen Pharmaceutical Companies of Johnson & Johnson, Turnhoutseweg 30, 2340 Beerse, Belgium; ^6^Janssen Prevention Center, Janssen Pharmaceutical Companies of Johnson & Johnson, 3210 Merryfield Row, San Diego, CA 92121, USA.

Running title: Functionally improved anti-PHF antibody

^#^These authors contributed equally

*To whom correspondence should be addressed: [AApetri@its.jnj.com](mailto:AApetri@its.jnj.com)

**Table S1** Overview of mutations investigated during the maturation process.

| **Mutation** | **Chain** | **Approach** | **Mutation** | **Chain** | **Approach** |
| --- | --- | --- | --- | --- | --- |
| hCBTAU-22.1(S30N) | VH | Random library | hCBTAU-22.1(H31Y) | VL | Random library |
| hCBTAU-22.1(V34L) | VH | Random library | hCBTAU-22.1(L30W) | VL | Random library |
| hCBTAU-22.1(V34M) | VH | Random library | hCBTAU-22.1(S57R) | VL | Random library |
| hCBTAU-22.1(S52R) | VH | Random library | hCBTAU-22.1(N33F) | VH | Rational Design |
| hCBTAU-22.1(G57V) | VH | Random library | hCBTAU-22.1(R50F) | VH | Rational Design |
| hCBTAU-22.1(T58I) | VH | Random library | hCBTAU-22.1(R50Y) | VH | Rational Design |
| hCBTAU-22.1(G103V) | VH | Random library | hCBTAU-22.1(R50W) | VH | Rational Design |
| hCBTAU-22.1(T104S) | VH | Random library | hCBTAU-22.1(D102A) | VH | Random library |
| hCBTAU-22.1(A109T) | VH | Random library | hCBTAU-22.1(C101S) | VH | Random library |
| hCBTAU-22.1(Q27R) | VL | Random library | hCBTAU-22.1(C101F) | VH | Random library |
| hCBTAU-22.1(H31L) | VL | Random library | hCBTAU-22.1(C101Y) | VH | Random library |
| hCBTAU-22.1(L97F) | VL | Random library | hCBTAU-22.1(C106W) | VH | Random library |
| hCBTAU-22.1(A109G) | VH | Random library | hCBTAU-22.1(R32P) | VL | Random library |
| hCBTAU-22.1(D102G) | VH | Random library | hCBTAU-22.1(S33P) | VL | Random library |
| hCBTAU-22.1(N33H) | VH | Random library | hCBTAU-22.1(H35N) | VL | Random library |
| hCBTAU-22.1(P53R) | VH | Random library | hCBTAU-22.1(H35D) | VL | Random library |
| hCBTAU-22.1(S30R) | VH | Random library | hCBTAU-22.1(L55W) | VL | Random library |
| hCBTAU-22.1(Y32F) | VH | Random library | hCBTAU-22.1(Q98R) | VL | Random library |

**Table S2** Names and sequences of tau peptides used in this study.

| Name | Residues^1^ | Amino acid sequence^2^ |
| --- | --- | --- |
| V1090-1 | 36-57 | EGDTDAGLKE**S**PLQ**T**PTEDGSE |
| V1085-1 | 103-120 | AEEAGIGDTP**S**LEDEAAG |
| V1089-10 | 187-205 | EPPKSGDRSG**YS**SPGSPGT |
| V1088-3 | 187-212 | EPPKSGDRSG**Y** SSPGSP G**T**PGSRSRT |
| V1091-5 | 192-212 | GDRSGYSSPG**S**PG**T**PGSRSRT |
| V1088-7 | 221-245 | REPKKVAVVR**T**PPKSPS**S**AKSRLQT |
| V1090-3 | 221-242 | REPKKVAVVR**T**PPK**S**PSSAKSR |
| V1088-32 | 386-407 | TDHGAEIVYK**S**PVV**S**GDTSPRH |
| V1088-5 | 404-429 | SPRHLSNVSS**T**GSIDMVD**S**PQLATLA |
| V1088-23 | 406-429 | RHLSNVSSTG**S**IDMVD**S**PQLATLA |
| V1089-24 | 412-429 | SSTGSIDMVD**S**PQLATLA |
| CL22D-P | 417-441 | (C)IDMVD**S**PQLATLADEVSASLAKQGL(EPEA)^3^ |

^1^ Number corresponding to residue numbering in 2N4R tau

^2^ Phosphorylated residues are indicated in bold and underlined

^3^ Amino acids shown in the brackets do not belong to the original tau sequence and are inserted to facilitate chemical ligation

**Table S3 Data collection for Fab-CBTAU-22.1 in complex with peptide V1088-5 and Fab-dmCBTAU-22.1 in complex with peptide V1088-23**

| Peptide  Fab | V1088-5  CBTAU-22.1 | V1088-23  dmCBTAU-22.1 |
| --- | --- | --- |
| X-ray source | PXI/X06SA (SLS^1^) | PXII/X10SA (SLS^1^) |
| Wavelength [Å] | 1.0000 | 0.9999 |
| Detector | EIGER X 16M | PILATUS 6M |
| Temperature [K] | 100 | 100 |
| Space group | P 2_1_ | P 2_1_ |
| Cell: a; b; c; [Å] | 108.06; 96.21; 108.98 | 107.58; 95.55; 108.34 |
| α; β; γ; [◦] | 90.0; 112.8; 90.0 | 90.0; 112.7; 90.0 |
| Resolution [Å] | 2.63 (2.88-2.63) | 1.95 (2.20-1.95) |
| Unique reflections | 59335 (14178) | 141912 (43522) |
| Multiplicity | 2.8 (2.8) | 2.8 (2.9) |
| Completeness [%] | 96.5 (97.4) | 96.2 (97.6) |
| R_sym_ [%]^3^ | 8.4 (43.5) | 6.4 (47.8) |
| R_meas_ [%]^4^ | 10.5 (53.4) | 7.9 (58.8) |
| Mean(I)/sd^5^ | 9.97 (2.70) | 12.20 (2.44) |

^1^ SWISS LIGHT SOURCE (SLS, Villigen, Switzerland)

^2^ values in parenthesis refer to the highest resolution bin.

^3^ with ^^where *I_h,i_* is the intensity value of the *i*th measurement of *h*

^4^ with ^^where *I_h,i_* is the intensity value of the *i*th measurement of *h*

^5^ calculated from independent reflections

**Table S4 Refinement statistics for Fab-CBTAU-22.1 in complex with peptide V1088-5 and Fab-dmCBTAU-22.1 in complex with peptide V1088-23**

| Peptide  Fab | V1088-5  CBTAU-22.1 | V1088-23  dmCBTAU-22.1 |
| --- | --- | --- |
| Resolution [Å] | 100.46-2.63 | 99.98-1.95 |
| Number of reflections (working /test) | 58513 / 821 | 139011 / 1923 |
| R_cryst_ [%] | 21.6 | 22.3 |
| R_free_[%]^2^ | 27.0 | 28.4 |
| Total number of atoms: |  |  |
| Protein | 13264 | 13344 |
| Water | 45 | 769 |
| Peptide | 246 | 270 |
| Deviation from ideal geometry: ^3^ |  |  |
| Bond lengths [Å] | 0.006 | 0.007 |
| Bond angles [°] | 1.24 | 1.43 |
| Bonded B’s [Å^2^] ^4^ | 5.6 | 3.1 |
| Ramachandran plot: ^5^ |  |  |
| Most favoured regions [%] | 92.0 | 93.4 |
| Additional allowed regions [%] | 8.0 | 6.6 |
| Generously allowed regions [%] | 0.0 | 0 |
| Disallowed regions [%] | 0.0 | 0 |
|  |  |  |

^1^ Values as defined in REFMAC5, without sigma cut-off
^2^ Test-set contains 1.4% of measured reflections
^3^ Root mean square deviations from geometric target values
^4^ Calculated with MOLEMAN
^5^ Calculated with PROCHECK

**Figure S1 Binding affinities of the antigen binding domains of CBTAU-22.1 (left) and dmCBTAU-22.1 (right) to biotinylated peptide V1089-24 as determined by Octet Biolayer Interferometry.** Binding curves for different concentrations of Fab were determined where equilibrium was reached in the association step. The range of Fab concentration tested was between 0-100 µM and 0-15 µM, respectively. The last 5 seconds of the association step was averaged as response and plotted against the corresponding Fab concentrations. This was then fit to a model to obtain the affinities via steady state analysis.

**Figure S2** Association and dissociation kinetics for the binding of CBTAU-22.1 (left) and dmCBTAU-22.1 (right) to peptide V1089-24 at different ionic strengths**.** Both CBTAU-22.1 and dmCBTAU-22.1 display a salt dependent binding profile, in line with the structural data, suggesting that their interaction with tau is driven mainly by electrostatic interactions. dmCBTAU-22.1 shows significantly improved binding at all tested salt concentrations.

**Figure S3** Association and dissociation kinetics for the binding of CBTAU-22.1 (left) and dmCBTAU-22.1 (right) to a panel of phosphorylated tau-derived peptides**.** Neither of the antibodies shows unspecific binding to tau peptides that do not contain the pSer^422^ epitope.


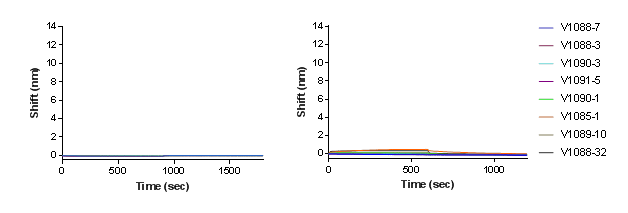


**Figure S4 Western blot detection of PHFs using anti-tau antibodies hTau10 (left) and AT8 (right).** PHF stocks were diluted 1:10 and a range of 20-2.5 μl of diluted PHF-tau is loaded on different lanes for reference and the experimental PHF batches, respectively. Both batches show the PHF characteristic band pattern for the entire concentration range. hTau10 is a pan tau antibody that binds both monomeric and PHF-tau whereas AT8 exclusively detects phosphorylated tau.


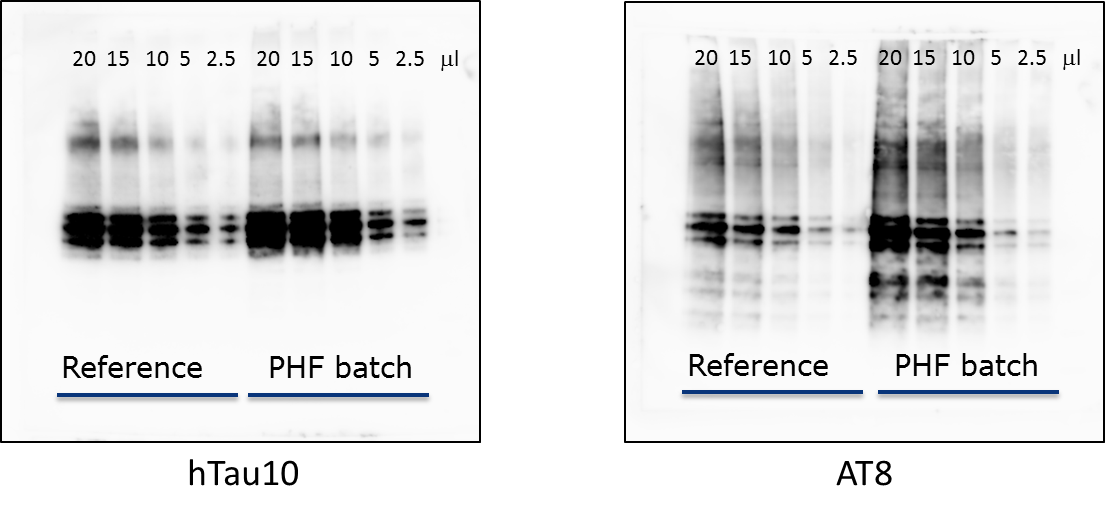

Supplement: Supplementary file 1 — Table S1. Overview of mutations investigated during the maturation process. Table S2. Names and sequences of tau peptides used in this study. Table S3. Data collection for Fab-CBTAU-22.1 in complex with peptide V1088-5 and Fab-dmCBTAU-22.1 in complex with peptide V1088-23. Table S4. Refinement statistics for Fab-CBTAU-22.1 in complex with peptide V1088-5 and Fab-dmCBTAU-22.1 in complex with peptide V1088-23. Figure S1. Binding affinities of the antigen binding domains of CBTAU-22.1 (left) and dmCBTAU-22.1 (right) to biotinylated peptide V1089-24 as determined by Octet Biolayer Interferometry. Figure S2. Association and dissociation kinetics for the binding of CBTAU-22.1 (left) and dmCBTAU-22.1 (right) to peptide V1089-24 at different ionic strengths. Figure S3. Association and dissociation kinetics for the binding of CBTAU-22.1 (left) and dmCBTAU-22.1 (right) to a panel of phosphorylated tau-derived peptides. Neither of the antibodies shows unspecific binding to tau peptides that do not contain the pSer422 epitope. Figure S4. Western blot detection of PHFs using anti-tau antibodies hTau10 (left) and AT8 (right). (DOCX 2093 kb) [file 40478_2018_562_MOESM1_ESM.docx]
